# Resistance of Gram-Negative Bacteria to Cefepime-Enmetazobactam: A Systematic Review

**DOI:** 10.3390/pathogens14080777

**Published:** 2025-08-06

**Authors:** Matthew E. Falagas, Laura T. Romanos, Dimitrios S. Kontogiannis, Katerina Tsiara, Stylianos A. Kakoullis

**Affiliations:** 1 Alfa Institute of Biomedical Sciences (AIBS), 9 Neapoleos Street, Marousi, 151 23 Athens, Greece; l.romanos@aibs.gr (L.T.R.); d.kontogiannis@aibs.gr (D.S.K.);; 2 School of Medicine, European University Cyprus, 6 Diogenous Str., 2404 Nicosia, Cyprus; s.kakoullis@euc.ac.cy; 3 Department of Medicine, Tufts University School of Medicine, 145 Harrison Ave, Boston, MA 02111, USA

**Keywords:** cefepime, enmetazobactam, cefepime-enmetazobactam, β-lactamases, metallo-β-lactamases, Enterobacterales, lactose non-fermenting Gram-negative, *Klebsiella pneumoniae*, *Escherichia coli*, *Pseudomonas aeruginosa*, *Acinerobacter baumannii*

## Abstract

Cefepime-enmetazobactam is a novel β-lactam/β-lactamase inhibitor combination showing good activity against multidrug-resistant (MDR) Gram-negative bacteria producing a variety of β-lactamases. In this systematic review, we aimed to evaluate the available data on resistance to this drug. We performed a thorough search of four databases (Embase, PubMed, Scopus, and Web of Science), as well as backward citation searching, to identify studies containing data on resistance to cefepime-enmetazobactam. The data were extracted and analyzed according to the breakpoints established by the European Committee on Antimicrobial Susceptibility Testing (EUCAST) and the Food and Drug Administration (FDA), or the specific breakpoints reported by the authors of the respective studies. Analysis based on the type of lactamases produced by the isolates was also performed. Ten studies reported in vitro susceptibility testing and mechanisms of antimicrobial resistance. The total number of isolates was 15,408. The activity of cefepime-enmetazobactam against β-lactamase-producing isolates was variable. The resistance of the studied extended-spectrum β-lactamase (ESBL)-producing and ampicillin C β-lactamase (AmpC)-producing isolates was low (0–2.8% and 0%, respectively). The resistance was higher among oxacillinase-48 β-lactamase (OXA-48)-producing and *Klebsiella pneumoniae* carbapenemase (KPC)-producing isolates (3.4–13.2% and 36.7–57.8%, respectively). High resistance was noted among metallo-β-^lactamase^ (MBL)-producing isolates (reaching 87.5% in one study), especially those producing New Delhi metallo-β-lactamase (NDM) and Verona integron-encoded metallo-β-lactamase (VIM), which had the highest rates of resistance. The high activity of cefepime-enmetazobactam against Enterobacterales and selected lactose non-fermenting Gram-negative pathogens, including ESBL-producing and AmpC-producing isolates, makes it a potential carbapenem-sparing agent. The drug should be used after in vitro antimicrobial susceptibility testing in patients with infections caused by OXA-48, KPC, and MBL-producing isolates.

## 1. Introduction

As Gram-negative pathogens continue to acquire mechanisms of antimicrobial resistance, there is a need for the development of new drugs. Among the various antimicrobial classes, β-lactams are the oldest class of antibiotics encompassing penicillins, cephalosporins, monobactams, and carbapenems [1,2]. Cefepime, a fourth-generation cephalosporin, has a broad spectrum of activity against Gram-negative and Gram-positive pathogens. It is stable against class C [such as ampicillin C β-lactamase (AmpC)], β-lactamases, and class D [such as oxacillinase-48 β-lactamase (OXA-48-like)] β-lactamases [3]. The in vitro activity of cefepime against extended-spectrum β-lactamase (ESBL) producers is rare [4]. To extend the spectrum of activity, cefepime is combined with β-lactamase inhibitors, namely enmetazobactam, and agents under investigation, including zidebactam and taniborbactam. Enmetazobactam is a novel penicillanic acid sulfone β-lactamase inhibitor that exhibits effectiveness against class A β-lactamases such as ESBLs, β-lactamases resistant to clavulanic acid, and carbapenemases [3].

Cefepime-enmetazobactam has been approved, as of 2024, for the treatment of patients with complicated urinary tract infections (cUTIs) and pyelonephritis in both Europe [by the European Medicines Agency (EMA)] and the US [by the Food and Drug Administration (FDA)], as well as for hospital-acquired pneumonia (HAP), including ventilator-associated pneumonia (VAP), and associated bacteremia in Europe [5,6]. The FDA advises that the drug be used to treat infections proven or strongly suspected to be caused by bacteria susceptible to it [5].

The pharmacokinetic data of cefepime-enmetazobactam support its use in patients with HAP, including VAP, and cUTIs. Both agents showed good distribution into the pulmonary epithelial lining fluid (ELF) [7]. In healthy volunteers, the mean percentage of partitioning of the total drug concentration between the ELF and blood plasma was 60.6% for cefepime and 53.0% for enmetazobactam, indicating considerable penetration of the lung tissue. This data supports the consideration of this agent for the treatment of pneumonia caused by MDR bacteria [8]. Additionally, cefepime and enmetazobactam are renally excreted, with a large percentage of both agents being recoverable unchanged in the urine (88% and 90%, respectively). Thus, they achieve high urinary concentrations, making them particularly effective for the treatment of patients with cUTIs and pyelonephritis [9].

In this systematic review, we performed an evaluation of the current evidence from in vitro susceptibility testing studies on the resistance of Gram-negative bacteria, including Enterobacterales and lactose non-fermenting Gram-negative bacteria, to cefepime-enmetazobactam. Such data may help physicians in the appropriate use of this new antibiotic in clinical practice.

## 2. Methods

### 2.1. Sources and Eligibility Criteria

We conducted a thorough literature review across four databases, specifically Embase, PubMed, Scopus, and Web of Science, from their inception to June 2025. Additionally, we performed backward citation searching to obtain as many studies as possible. Eligible for assessment were studies of any primary research design that met the following inclusion criteria: (a) the terms cefepime-enmetazobactam included in the title/abstract/keywords, and (b) the terms minimum inhibitory concentration (MIC) or disk diffusion susceptibility testing data present.

The exclusion criteria were (a) non-primary research articles; (b) articles involving studying isolates acquired from animal samples; (c) case reports of a single isolate or patient; (d) primary research articles that did not contain relevant data for this review; (e) conference abstracts; (f) studies evaluating less than 10 isolates for the cefepime-enmetazobactam susceptibility testing.

### 2.2. Search Strategy

The detailed search strategy is presented in Supplementary Table S1. Terms such as “cefepime-enmetazobactam”, “resistance”, “non-susceptibility”, “MIC”, and “disk diffusion” were used. Additional articles were identified through manual screening of reference lists.

### 2.3. Screening of Studies

We performed deduplication of studies from different databases using the automatic deduplication feature of the Rayyan tool, which utilized the digital object identifiers (DOIs) of the studies. We screened all the retrieved records using the full text.

### 2.4. Breakpoints of Susceptibility Testing

Breakpoints of antimicrobial resistance to cefepime-enmetazobactam were based on published guidelines for Enterobacterales by the European Committee on Antimicrobial Susceptibility Testing (EUCAST) (cefepime MIC > 4 mg/L with a fixed concentration of enmetazobactam) and the FDA (cefepime MIC > 16 mg/L with a fixed concentration of enmetazobactam). As of 2019, the EUCAST altered the susceptibility categories from “susceptible, intermediate, and resistant” to “susceptible and resistant”. The susceptible category includes the subcategory called “susceptible, increased exposure”. At the time of writing (June 2025), the Clinical and Laboratory Standards Institute (CLSI) has not yet established breakpoints for this antibiotic combination. For older studies, performed before the establishment of EUCAST and FDA breakpoints for resistance to the combination of cefepime and enmetazobactam, the 34th edition of CLSI M100 breakpoints for resistance to cefepime were used (MIC ≥ 16 mg/L) [10].

### 2.5. Data Extraction

In our analysis, we included data on the number of total studied isolates, the number of isolates of specific species, and the presence of various β-lactamases (based on phenotypic and/or genotypic methods). Additionally, we included data on the MIC range, MIC_50_, MIC_90_, and the percentage of resistance of the studied isolates. The study selection and screening, as well as data extraction, were conducted independently by two investigators (L.T.R. and D.S.K.). Discrepancies were discussed with a senior investigator (M.E.F.).

### 2.6. Data Tabulation

The data were organized in the table according to bacterial species and β-lactamase production. The following data were collected: specific isolates, production of β-lactamases, MIC range in mg/L, and proportion of resistance to cefepime-enmetazobactam based on the authors’ criteria, as defined in each study (according to the EUCAST, CLSI, or FDA MIC susceptibility breakpoints).

## 3. Results

### Selection of Relevant Articles

**Figure 1:**
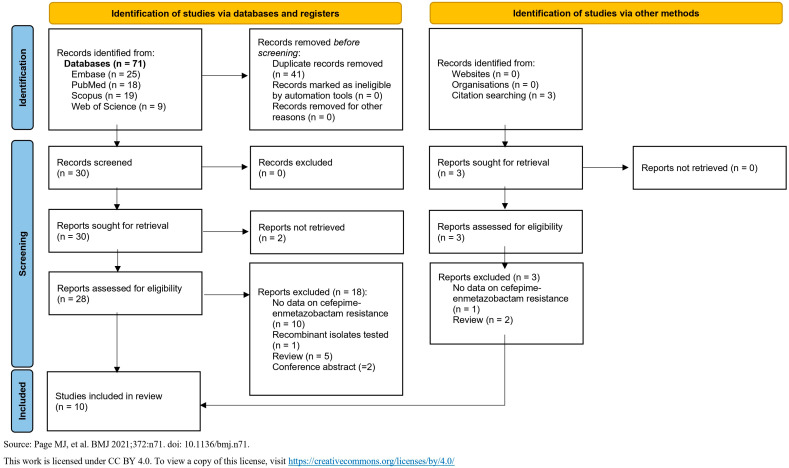
“Preferred Reporting Items for Systematic Reviews and Meta-Analyses” (PRISMA) flow diagram for identifying, screening, and selecting articles. (Source: [11]).

This systematic review was conducted in accordance with the Preferred Reporting Items for Systematic Reviews and Meta-Analyses (PRISMA) guidelines. The PRISMA reporting checklist for the abstract and the text is shown in Supplementary Tables S2 and S3. The PRISMA flow diagram, which describes the evaluation, selection, and inclusion of relevant articles, is shown in Figure 1. In total, we identified 71 articles, and after deduplication, 30 were selected for screening. A comprehensive text evaluation was conducted for all 30 articles, resulting in the exclusion of 20 of them.

In total, 10 in vitro studies were included in this analysis, published between 2019 and 2025 (Table 1) [12,13,14,15,16,17,18,19,20,21]. The studies included 15,408 isolates, comprising Enterobacterales and lactose non-fermenting Gram-negative bacteria. Nine studies reported in detail the isolates that were tested, whereas one study reported that only carbapenem-resistant Enterobacterales were evaluated. Specifically, the pathogens were *Escherichia coli* (eight studies), *Klebsiella pneumoniae* (seven studies), *Enterobacter cloacae* (four studies), *Citrobacter freundii* (three studies), *Citrobacter koseri* (three studies), *Klebsiella aerogenes* (three studies), *Klebsiella oxytoca* (three studies), *Serratia marcescens* (three studies), *Proteus mirabilis* (two studies), *Providencia stuartii* (two studies), *Pseudomonas aeruginosa* (two studies), *Acinetobacter baumannii* (one study), *Citrobacter sedlakii* (one study), *Klebsiella quasipneumoniae* (one study), *Morganella morganii* (one study), and *Providencia rettgeri* (one study) (Table 1).

Several breakpoints were applied in the studies included in this article. Some authors used the EUCAST resistance breakpoint for cefepime-enmetazobactam in Enterobacterales (MIC > 4 mg/L), while others used the FDA resistance breakpoint for cefepime-enmetazobactam in Enterobacterales (MIC > 16 mg/L). Older studies, performed before the establishment of breakpoints for the combination of cefepime-enmetazobactam, utilized the cefepime resistance breakpoints as per CLSI (MIC ≥ 16 mg/L). One study, which included *P. aeruginosa* and *A. baumannii* isolates, used a projected breakpoint determined by pharmacokinetic/pharmacodynamic (PK/PD) data [18]. EUCAST resistance breakpoints for cefepime in Enterobacterales (2019 and 2020) (MIC > 4 mg/L) were also utilized in some studies. Combinations of the various breakpoints were also employed in some studies.

According to the results of the included studies, cefepime-enmetazobactam has variable resistance proportions among the tested isolates, with MICs ranging from ≤0.015 mg/L to> 64 mg/L (Table 1). The presence of specific β-lactamases affects the susceptibility of bacteria to this drug combination. Specifically, the proportion of resistance of ESBL-producing Enterobacterales ranges from 0% to 2.8% in relevant studies [12,16,19,21,22]. The proportion of resistance of AmpC-producing was 0% among 85 Enterobacterales in one relevant study. Three studies reported 3.4%, 1.2%, and 13.2% resistance among 119, 1000, and 304 isolates carrying OXA-like enzymes, respectively, and one reported 67% resistance among 33 OXA-like enzyme producers [13,14,15,21]. Resistance of KPC-producing isolates ranged from 36.7% to 57.8% [13,14,15,20]. Higher proportions of resistance were noted in MBL-producing bacteria compared to bacteria producing class A (such as ESBLs or KPC) or class C (such as AmpC) β-lactamases. In one study, the MBL-producing isolates had a higher proportion of resistance (87.5%) compared to KPC-producing (56.8%) or OXA-48-producing (13.2%) isolates (Table 1) [14]. Additionally, in another study, 99.1% of 601 NDM-producing isolates and 39.4% of 178 VIM-producing isolates were resistant to cefepime-enmetazobactam, compared to 36.7% of 51 KPC-producing and 1.2% of 1000 OXA-48-like-producing isolates (Table 1) [13].

## 4. Discussion

In our thorough analysis, we evaluated the activity of a combination of cefepime and enmetazobactam against Enterobacterales, as well as lactose non-fermenting Gram-negative bacteria, specifically *Acinetobacter baumannii* and *Pseudomonas aeruginosa*. We highlighted the low resistance of bacteria, especially ESBL and AmpC-producing Gram-negative bacteria, to this drug and analyzed the mechanisms of resistance in isolates that were β-lactamase producers. The results of the included studies demonstrated that cefepime-enmetazobactam was less effective against OXA-48, KPC, and even more so MBL-producing isolates, which cause infections that have disseminated worldwide [23]. This drug combination has low activity against MBLs because enmetazobactam is a serine β-lactamase inhibitor and does not display inhibitory activity against MBL-producing isolates [24]. This is explained by the fact that MBLs are zinc-dependent enzymes that are not inhibited by serine-based β-lactamase inhibitors, like tazobactam, enmetazobactam, relebactam, or avibactam. The findings from our analysis will help clinicians make informed decisions when using this new antibiotic. The data suggest that treating patients with cefepime-enmetazobactam is more effective based on the in vitro antimicrobial susceptibility testing, which helps avoid treatment failure, especially in infections caused by OXA-48-, KPC-, and MBL-producing isolates.

This data is further supported by an additional study, excluded from our table because of a low number of isolates [25]. This study analyzed the MIC modal values of 9 ESBL-producing *Klebsiella pneumoniae* isolates and showed an MIC range of 0.06 to 2 mg/L, highlighting the effectiveness of this antibiotic combination against such isolates [25].

Our data showed that cefepime-enmetazobactam has good potential as a carbapenem-sparing agent due to its high activity against bacteria producing a variety of β-lactamases, including ESBLs and AmpCs [9]. Other β-lactam/β-lactamase inhibitor combinations that may be used as carbapenem-sparing agents include newer combinations, including ceftazidime-avibactam and ceftolozane-tazobactam, as well as fosfomycin [26,27,28]. More specifically, ceftazidime-avibactam showed non-inferiority compared to carbapenems (specifically meropenem) for patients with complicated intra-abdominal infections (cIAIs), HAP, or VAP caused by Gram-negative bacteria [29,30]. In addition, ceftolozane-tazobactam showed non-inferiority compared to carbapenems (specifically meropenem) for the treatment of patients with cIAIs or VAP [31,32].

Intravenous fosfomycin is also active against a considerable proportion of ESBL-producing and extensively drug-resistant (XDR) bacteria [27,28,33,34]. A total of 81% of 2416 *Escherichia coli* isolates from patients in India, of which 47.6% were ESBL-producing, demonstrated susceptibility to fosfomycin in an in vitro antimicrobial susceptibility study [33]. In addition, a study examined the in vitro antimicrobial susceptibility of a total of 152 MDR Enterobacterales isolates from patients in Greece, including *Klebsiella pneumoniae* (76.3%), *Escherichia coli* (17.1%), *Proteus mirabilis* (4.6%), and other species (2.0%). Susceptibility to fosfomycin was 94.9%, 94.1% and 83.3% for carbapenemase-producing, ESBL-producing, and MBL-producing isolates, respectively [34]. These data, in conjunction with those from a systematic review of the literature, provide support for considering intravenous fosfomycin as a carbapenem-sparing agent for infections caused by ESBL-producing bacteria [27,28].

Additionally, two investigational combinations, namely cefepime-zidebactam and cefepime-taniborbactam, may be shown to be effective in treating patients with carbapenem-resistant infections, as data suggest that these agents exhibit effectiveness against carbapenem-resistant isolates [35,36]. Cefepime is a fourth-generation cephalosporin that acts by binding to penicillin-binding proteins (PBPs) [37]. Zidebactam is a new β-lactamase inhibitor. Besides its activity in inhibiting the hydrolysis of β-lactams by β-lactamases, the new drug enhances the antimicrobial activity of cefepime by binding to PBPs, particularly PBP2 [38]. Cefepime-zidebactam is currently in the late stages of clinical development, including a Phase 3 clinical trial (with registration code NCT04979806/W-5222-301) [39]. Taniborbactam is also a new β-lactamase inhibitor, specifically a cyclic boronate derivative, that inhibits the hydrolysis of cefepime and thereby restores its activity [37]. Currently, taniborbactam is being studied in clinical trials of patients with cUTIs [40].

Cefepime-enmetazobactam has already been approved for the treatment of patients with cUTIs and pyelonephritis in both Europe and the US, as well as for HAP, including VAP, and associated bacteremia in Europe [5,6]. A Phase 3 clinical trial (NCT03687255) conducted by “Allecra Therapeutics” demonstrated that cefepime-enmetazobactam had a statistically significantly higher rate of patients in the primary analysis set who achieved overall treatment success, encompassing both clinical cure and microbiological eradication of the infection (defined as bacterial count <10^3^ CFU/mL in urine culture), compared to piperacillin-tazobactam (79.1% versus 58.9%) [41]. Several other Phase 1 (NCT03775668, NCT03680352, NCT03680378, NCT03685084) and Phase 2 (NCT03680612, NCT05826990) clinical trials have been conducted to study the safety and pharmacokinetic profile of cefepime-enmetazobactam in adults [42,43,44,45,46,47]. More recently, a Phase 2 trial was initiated (NCT05826990) to evaluate the use of cefepime-enmetazobactam in pediatric patients with cUTIs, with the aim of extending the indication of this drug to children. At the time of writing this article, the status of this trial is still “recruiting” [47].

In a preclinical study, mice treated with the drug combination showed a significant decrease in bacterial counts in the lungs when compared to untreated controls over a 26 h period [48]. This effect was consistent across all the tested strains and was strongest against the strain with the highest MIC value (8 mg/L) [48]. Bacterial stasis of growth was observed during the comparison of bacterial populations from the 2nd to the 26th hour after treatment [48]. This data supports cefepime-enmetazobactam’s retained activity against resistant bacterial strains. Additionally, in another in vivo study in a neutropenic murine pneumonia model, the drug exhibited favorable pharmacokinetic and pharmacodynamic characteristics, supporting the use of cefepime 2 g and enmetazobactam 0.5 g dosages in humans with cUTIs or pyelonephritis [7].

There is limited data on the adverse events associated with cefepime-enmetazobactam, primarily due to the short duration of clinical use after approval by the EMA and FDA. According to the EMA and the FDA, common adverse events (occurring in 1 out of 10 people receiving the drug) associated with cefepime-enmetazobactam are high levels of transaminases [alanine aminotransferase (ALT) and aspartate aminotransferase (AST)] and bilirubin, diarrhea, headache, skin rash, and phlebitis at the injection site [5,6]. Uncommon adverse reactions (occurring in up to 1 in 100 people) include *Clostridium difficile*-associated diarrhea, inflammation of the large intestine, oral fungal infections, and vaginal infections, among others [5,6]. Other, rare adverse events (occurring in 1 out of 1000 patients) include shortness of breath, abdominal pain, convulsions, and dysgeusia [5,6]. Contraindications to the use of cefepime-enmetazobactam, according to the EMA, include patients with hypersensitivity to cephalosporins or other β-lactam antibiotics [6].

This study has some limitations. Despite the thorough evaluation of the available literature, 10 in vitro studies were selected for inclusion. Additionally, each study had heterogeneous data, including different isolates and β-lactamases detected, as well as varying susceptibility breakpoints. The heterogeneity between studies on the above factors does not allow for the synthesis of the available evidence using the statistical techniques of meta-analysis. Additionally, no risk of bias assessment was conducted for the studies included in our analysis, as there is no generally accepted and validated tool for assessing risk of bias in in vitro antimicrobial susceptibility studies. Thus, these limitations of the analyzed data preclude a robust conclusion.

## 5. Conclusions

The published data demonstrate that cefepime-enmetazobactam has high in vitro antimicrobial activity against most studied Gram-negative bacteria, especially ESBL and AmpC-producing isolates. However, the proportion of resistance among studied OXA-48-producing isolates is higher, and it is considerable for KPC-producing isolates. Additionally, the proportion of resistance among MBL-producing Gram-negative bacterial isolates is high. These data also suggest that in vitro antimicrobial susceptibility testing and molecular diagnostic testing for resistance mechanisms, when available, should be performed before administering cefepime-enmetazobactam to patients to improve clinical outcomes.

## Figures and Tables

**Table 1:** Proportion of resistance of various isolates to cefepime/enmetazobactam.

Author *	Year	Isolates	N	β-Lactamase Production (Number of Isolates)	MIC Value or Range (mg/L)	MIC_50_ (mg/L)	MIC_90_ (mg/L)	Resistance % (I: %) ^a^ [Breakpoint] ^b^
Emeraud [15]	2025	CREs OXA-48-like producers AmpC overproduction/ ESBL + decreased outer membrane permeability NDM KPC VIM more than one β-lactamase *K. pneumoniae* *E. coli* *E. cloacae* complex *C. freundii* *K. aerogenes* *K. oxytoca* *C. koseri* *M. morganii* *S. marcescens* *P. mirabilis* *R. ornithinolytica*	291 119 97 45 18 6 6 91 69 67 23 14 14 5 4 2 1 1	NA Hcase or ESBL + imperm. ^e^ (25), OXA-48-like (34), NDM (18), KPC (16) Hcase or ESBL + imperm. (10), OXA-48-like (48), NDM (11), KPC (1) Hcase or ESBL + imperm. (38), OXA-48-like (12), NDM (12), VIM (6) Hcase or ESBL + imperm. (3), OXA-48-like (1), NDM (5), VIM (1) Hcase or ESBL + imperm. (12), OXA-48-like (1), NDM (1) Hcase or ESBL + imperm. (6), OXA-48-like (7), KPC (1) Hcase or ESBL + imperm. (2), OXA-48-like (3) Hcase or ESBL + imperm. (1), OXA-48-like (1), NDM (2) OXA-48-like (1), NDM (1) OXA-48-like (1) OXA-48-like (1)	≤0.06–>16 ≤0.06–>16 ≤0.06–>16 4–>16 2–>16 1–>16 >16 ≤0.06–>16 ≤0.06–>16 ≤0.06–>16 ≤0.06–4 ≤0.06–>16 ≤0.06–8 ≤0.06–0.5 ≤0.06–8 0.12–8 ≤0.06 0.25	NA 0.12 2 >16 4 2 >16 0.12 >16 2 0.5 0.5 0.12 4 >16 1 NA NA	NA 1 8 >16 >16 >16 >16 1 >16 8 1 1 0.25 8 >16 2 NA NA	26.5 [EUCAST] ^c^, 18.2 [FDA] ^d^ 3.4 [EUCAST], 1.7 [FDA] 16.5 [EUCAST], 10.1 [FDA] 95.6 [EUCAST], 91.1 [FDA] 38.9 [EUCAST], 11.1 [FDA] 16.7 [EUCAST], 16.7 [FDA] 100 [EUCAST], 100 [FDA] 7.7 [EUCAST], 5.5 [FDA] 75.4 [EUCAST], 55 [FDA] 20.9 [EUCAST], 1.5 [FDA] 0 [EUCAST], 0 [FDA] 0 [EUCAST], 0 [FDA] 0 [EUCAST], 0 [FDA] 20 [EUCAST], 0 [FDA] 75 [EUCAST], 75 [FDA] 0 [EUCAST], 0 [FDA] 0 [EUCAST], 0 [FDA] 0 [EUCAST], 0 [FDA]
Bonnin [13]	2024	CREs Total carbapenemase producers OXA-48-like producers NDM producers VIM producers KPC producers Non-carbapenemase producers	2212 2089 1000 601 178 51 123	OXA-48 (721), NDM-1 (325), NDM-5 (260), OXA-181 (133), OXA-244 (109), VIM-1 (88), VIM-4 (88), NDM-7 (65), KPC-3 (43), NDM-14 (36), CASE (31), ESBL (27), CASE ACQ (17), OXA-484 (18), HYPER SHV-1 (11), OXA-204 (10), NDM-4 (7), OXA-232 (7), KPC-2 (6), IMI-1 (3), HYPER OXA-10 (2), HYPER OXY (2), IMI-2 (2), IMI-6 (2), NDM-19 (2), NDM-9 (2), VIM-12 (2), VIM-19 (2), IMP-22 (1), HYPER OXA-1 (1), HYPER TEM-1 (1), IMI-4 (1), IMI-19 (1), KPC-130 (1), KPC-31 (1), OXA-1181 (1), OXA-162 (1).	NA ≤0.25–>16 4–>16 4–>16 1–>16 ≤0.25–>16 ≤0.25–>16	NA NA ≤0.25 16 8 2 2	NA 1 2 >16 >16 >16 >16	NA ^c^ NA 1.2 99.1 39.4 36.7 21.5
Morrissey [19]	2024	Total isolates *E. coli* *K. pneumoniae* *E. cloacae* *K. oxytoca* *K. aerogenes* *C. freundii* *S. marcescens* *P. mirabilis* *P. rettgeri* *P. stuartii* Meropenem non-susceptible isolates *K. pneumoniae* *E. cloacae* *C. freundii* *K. aerogenes* *K. oxytoca* *S. marcescens* Total 3GC-R *E. coli* *K. pneumoniae* *E. cloacae* *C. freundii* Enterobacterales, ESBL gen. *K. pneumoniae*, ESBL gen. *E. coli*, ESBL gen. Enterobacterales, AmpC gen. *E. cloacae*, AmpC gen. *C. freundii*, AmpC gen. Enterobacterales, AmpC + ESBL gen.	2627 925 772 307 153 150 82 82 80 41 35 72 63 4 2 1 1 1 320 96 119 60 29 206 102 89 85 40 25 17	NA KPC-3 (36), CTX-M-1 (3), ACT (1), KPC-2 (1), OXA-48 (1), KPC-3 + CTX-M-1 (9), NDM-1 + CTX-M-1 (3), KPC-2 + CTX-M-1 (2), OXA-48 + CTX-M-1 (2), KPC-2 + SHV-ESBL (1), OXA-244 + CTX-M-1 (1), OXA-232 + CTX-M-1 (1), VIM-1 + ACT (1), VIM-1 + SHV-ESBL (1), VIM-1 + KPC-3 + CTX-M-1 (3), NDM-1 + CTX-M-1 + SHV-ESBL (1)VIM-1 + CTX-M-1 + KPC-27 (1), VIM-1 + ACT + SHV-ESBL (1), VIM-1 + CMY-2 + SHV-ESBL (1), VIM-1 + CMY-2 + CTX-M-9 + OXA-48 + SHV-ESBL (1) CTX-M-1 (183), CTX-M-9 (6), CTX-M-15 (2), SHV-ESBL (2), TEM-ESBL (1), CTX-M-1 + SHV-ESBL (7), CTX-M-9 + SHV-ESBL (2), CTX-M-1 + CTX-M-9 (1), CTX-M-1 + TEM-ESBL (1), SHV-ESBL + TEM-ESBL (1), ACT (38), CMY (30), MIR (9), DHA (6), ACC (1), CMY + CTX-M-1 (6), CTX-M-1 + OXA-48 (6), ACT + CTX-M-1 (5), MIR + CTX-M-1 (3), DHA + CTX-M-9 (2), ACT + CMY (1). DHA + CTX-M-1 (1), CTX-M-1 + OXA-48 + CMY (1), CTX-M-1 + TEM-ESBL + OXA-48 (1).	≤0.015–>64 ≤0.015–2 ≤0.015–>64 ≤0.015–>64 ≤0.015–32 ≤0.015–4 ≤0.015–64 ≤0.015–4 ≤0.015–0.25 ≤0.015–0.12 0.03–0.5 NA 0.03–64 0.03–2 0.03–32 0.06–64 0.06–16 0.03–2 0.03–2 0.03–2 0.03–4 0.12–2 0.06–1 0.06–1	0.03 0.03 0.03 0.12 0.03 0.06 0.06 0.12 0.06 ≤0.015 0.06 NA 0.06 0.06 0.06 0.5 0.25 0.06 0.06 0.06 0.25 0.5 0.25 0.12	0.25 0.06 0.5 1 0.06 0.25 0.5 0.25 0.06 0.03 0.25 NA 0.5 0.12 0.5 1 1 0.25 0.25 0.25 1 1 0.5 1	2.1 ^c^ 0 6.1 1.3 0.6 0 3.7 0 0 0 0 27.8 0.9 0 0.8 1.7 3.4 0 0 0 0 0 0 0
Kadry [16]	2022	*E. coli*	140	ESBL (65)	≥0.25–64	NA	NA	2.9 (I: 11.4) ^g^
Liu [18]	2022	Carbapenem non-susceptible *P. aeruginosa* *A. baumannii*	405 150 255	NA	NA 0.5–>64 ≤0.03–>64	NA 8 >64	NA 32 >64	NA 16 (I: 26) ^h^ NA
Vázquez-Ucha [14]	2022	Enterobacterales ^f^ No producing ESBLs Producing ESBLs OXA-48-producing isolates No producing ESBLs Producing ESBLs KPC-producing isolates No producing ESBLs Producing ESBLs MBL-producing isolates No producing ESBLs Producing ESBLs	400 106 294 304 57 247 44 27 17 56 24 32	CTXM-15 (358), OXA-1 (212), TEM-1 (196), SHV-11 (168), SHV-28 (85), KPC-3 (38), VIM-1 (40), SHV-12 (27), ACT-like (19), CTX-M9 (19), EC-like (17), ACT-24 (10), TEM-like (9), ACT-17 (8), ACT-16 (7), OXA-9 (7), OXY-2- like (7), SRT-like (7), SHV-76 (12), CMY-like (5), CTX-M1 (5), DHA-1 (5), NDM-1 (5), ACC-1 (5), KPC-2 (3), SHV-5 (3), TEM-40 (3), CTX-M14 (3), ACT-1 (2), E. coli AmpC β-lactamase (2), IMP-13 (2), OXA-2 (2), OXY-1-1 (2), OXY-2-7 (2), SHV-like (2), CMY-48 (2), NDM (1), NDM-23 (1), NDM-5 (1), NDM-7 (1), IMP-8 (1), SHV-155 (1), SHV-163 (1), SHV-55 (1), SHV-71 (1), TEM-163 (1), TEM-185 (1), TEM-95 (1), TEM112 (1), ACT-25 (1), CMY-117 (1), CMY-2 (1), CMY-75 (1), CTX-M58 (1), LEN-16 (1), MIR-like (4), OKP-B-like (1), OXA-10 (1), OXA162 (1), OXA320 (6), OXA663 (1), OXY-1- like (1), OXY-2-7 (1), OXY-6-like (1), OXY-6-2 (1)	≤0.5–≥128 ≤0.5–≥128 ≤0.5–≥128 NA	1 2 1 1 0.5 1 64 ≥128 ≤0.5 64 32 64	≥128 ≥128 32 16 4 16 ≥128 ≥128 1 ≥128 ≥128 ≥128	27.5, (I: 10.7) ^g^ 43.4(I: 5.7) 21.8(I: 12.5) 13.2 (I: 12.1) 5.3 (I: 7.0) 15 (I: 13.3) 56.8 (I: 2.3) 92.6 (I: 0) 0 (I: 5.9) 87.5 (I: 8.9) 83.3 (I: 8.4) 90.6 (I: 9.4)
Belley [12]	2021	Enterobacterales, all Enterobacterales, 3GC-R Enterobacterales, ESBL gen. *E. coli*, all *E. coli*, 3GC-R subgroup *E. coli*, ESBL gen. *K. pneumoniae* *K. pneumoniae*, 3GC-R *K. pneumoniae*, ESBL gen.	7168 1252 801 2516 451 418 2109 278 299	ESBLs (854): CTX-M type (622), SHV type (92), TEM type (14), VEB type (8) AmpC (448): ACT type (261), CMY type (166), MIR type (12), DHA type (26), FOX type (4), ACC (2) KPC (86): KPC-3 (75), KPC-2 (10), KPC-29 (1) OXA (51): OXA-48 (46), OXA232 (4), OXA-181 (1) MBL (31): VIM1 (17), NDM-1 (13), IMP-13 (1)	≤0.008–>64 ≤0.008–>64 0.015–32 ≤0.008–8 0.015–8 0.015–8 ≤0.008–>64 ≤0.008–32 0.015–32	0.03 0.12 0.06 0.03 0.06 0.06 0.03 0.06 0.06	0.25 0.5 0.5 0.03 0.25 0.25 0.25 0.25 0.5	1.7/1.2 [CLSI] ^g^, 2.3 [EUCAST] ^i^ 1.2/0.4 [CLSI], 2.9 [EUCAST] 1.1/0.1 [CLSI], 2.4 [EUCAST] 0.3/0 [CLSI], 0.4 [EUCAST] 1.1/0 [CLSI], 1.6 [EUCAST] 1/0 [CLSI], 1.4 [EUCAST] 3.9/3.1 [CLSI], 4.5 [EUCAST] 0.4/0 [CLSI], 0.4 [EUCAST] 1.3/0.3 [CLSI], 3.7 [EUCAST]
Lee [17]	2021	*E. coli* *K. pneumoniae*	26 175	KPC (69), OXA-48 like (12), NDM (4), VIM (4), OXA-48 like + KPC (1), OXA-48 like + NDM (1)	≤0.03–>64 ≤0.03–>64	1 4	4 >64	NA ^g^ NA
Tselepis [21]	2020	Total Enterobacterales KPC-positive ESBL-positive ^j^ OXA-positive ^k^ *K. pneumoniae* *E. coli* *E. cloaceae* *C. koseri* *C. sedlakii* *P. stuartii*	264 117 107 33 163 94 4 1 1 1	NA	NA	NA 32 ≤0.06 2 NA	NA 64 0.125 >64 NA	NA NA 2 67 NA
Morrissey [20]	2019	Total Enterobacterales *E. coli* *E. coli* ESBL gen. *K. pneumoniae* *K. pneumoniae* ESBL gen. ^l^ *K. pneumoniae* KPC gen. ^m^ *E. aerogenes* *E. cloacae* *P. aeruginosa*	1696 697 109 799 102 45 100 100 297	ESBL-positive *E. coli* (from 114 genotyped) ^n^: CTX-M (96), SHV (4), AmpC (2), KPC (2), TEM (1), VIM (1), CTX-M + CTX-M (2), TEM + CTX-M (1), KPC + CTX-M + AmpC (1), AmpC + CTX-M (4), SHV + AmpC (1) ESBL-positive *K. pneumoniae* (from 151 genotyped) ^n^: CTX-M (75), KPC (30), SHV (10), VIM (1), CTX-M + KPC (5), CTX-M + KPC + OXA (1), AmpC + CTX-M (1), OXA + CTX-M (12), CTX-M + SHV (2), CTX-M + SHV + OXA (2), KPC + SHV(8), KPC + SHV +OXA (1), VIM + SHV (1), OXA + SHV (2)	0.015–>64 0.015–32 0.015–32 0.015–>64 0.03–8 0.5–>64 0.015–2 0.03–>64 0.12–>64	0.06 0.06 0.06 0.06 0.12 16 0.06 0.12 4	0.25 0.12 0.12 0.5 1 >64 0.25 1 16	1.9 [EUCAST] ^i^, 1.5 [CLSI] ^g^ 0.1 [EUCAST], 0.1 [CLSI] 0.9 [EUCAST], 0.9 [CLSI] 3.6 [EUCAST], 2.8 [CLSI] 0 [EUCAST], 0 [CLSI] 57.8 [EUCAST], 42.2 [CLSI] 0 [EUCAST], 0 [CLSI] 3 [EUCAST], 2 [CLSI] NA

Notes: * Studies are presented in descending chronological order (and alphabetical order within a year); ^a^ I = intermediate susceptibility; ^b^ according to the criteria, as defined by the authors in each study. ^c^ EUCAST Susceptibility breakpoint MIC ≤ 4 mg/L, resistance breakpoint MIC > 4 mg/L.; ^d^ FDA susceptibility breakpoint MIC ≤ 8 mg/L., resistance breakpoint MIC > 16 mg/L.; ^e^ AmpC overproduction/ESBL + decreased outer membrane permeability; ^f^ five strains produced two carbapenemases: OXA-48 + KPC-3, OXA-48 + IMP-13, OXA-48 + NDM-1, OXA-48 1 + VIM-1, KPC-21 + IMP-22.; ^g^ CLSI cefepime susceptibility breakpoint MIC ≤ 2 mg/L, susceptible, dose-dependent breakpoint MIC ≤ 8 mg/L, resistance breakpoint MIC ≥ 16 mg/L.; ^h^ application of a breakpoint of MIC 8 mg/L (determined by PK/PD data); ^i^ EUCAST cefepime 2019 and 2020 susceptibility breakpoint of MIC ≤ 1 mg/L, resistance breakpoint MIC > 4 mg/L.; ^j^ ESBL strains consist of CTX-M-14- (48) and CTX-M-15- (66)- and TEM- (1)-positive strains. One strain was also positive for CMY-2b; ^k^ OXA-48/OXA-48-like. Three strains also carry VIM; ^l^ isolates containing an ESBL gene with or without OXA-48 and/or AmpC genes, ^m^ isolates containing a KPC gene with or without ESBL, OXA-48, and/or AmpC genes, ^n^ the β-lactamase genes identified included CTX-M-1, -9, -14, -15, -22, -27, -32, -61, -55, and -181, SHV-2, -2A, -7, -12, and -28, TEM-24 and -28, KPC-2 and -3, VIM-1, the AmpCs: CMY, ACC-1, DHA-7, and OXA-48 and -232. **Abbreviations:** 3GC-resistant, third-generation cephalosporin-resistant; ACC, Ambler class C β-lactamase (ACC-type); ACT, AmpC-type β-lactamase (ACT family); AmpC, ampicillin C β-lactamase; CASE, chromosomal AmpC β-lactamase; CASE ACQ, acquired AmpC β-lactamase; CLSI, Clinical and Laboratory Standards Institute; CMY, cephamycinase (CMY-type AmpC β-lactamase); CRE, carbapenem-resistant Enterobacterales; CTX-M, cefotaximase-Munich (CTX-M-type ESBL); DHA, Dhahran (DHA-type AmpC β-lactamase); EC-like, *Escherichia coli*-like AmpC β-lactamase; ESBL, extended-spectrum β-lactamase; ESBLg, extended-spectrum β-lactamase gene (s); EUCAST, European Committee on Antimicrobial Susceptibility Testing; FDA, U.S. Food and Drug Administration; FOX, cephamycinase (FOX-type AmpC β-lactamase); gen., generation; Hcase, hyperproduction of chromosomal AmpC β-lactamase; HYPER, hyperproduction; IMP, imipenemase (IMP-type metallo-β-lactamase); imperm., impermeability; IMI, imipenem-hydrolyzing β-lactamase (IMI-type); iESBL, isolate carrying extended-spectrum β-lactamase; KPC, Klebsiella pneumoniae carbapenemase; LEN, London enzyme β-lactamase; MBL, metallo-β-lactamase; MIR, AmpC-type β-lactamase (MIR family); NA, not applicable/not available; NDM, New Delhi metallo-β-lactamase; non-CPE, non-carbapenemase-producing Enterobacterales; OKP, other *Klebsiella pneumoniae* beta-lactamase; OXA, oxacillinase (OXA-type carbapenemase); OXY, Oxytoca β-lactamase (*Klebsiella oxytoca*); PK/PD, pharmacokinetic/pharmacodynamic; SHV, sulfhydryl variable β-lactamase; SRT-like, *Serratia fonticola*-like AmpC β-lactamase; TEM, Temoniera β-lactamase; VEB, Vietnamese extended-spectrum β-lactamase; VIM, Verona integron-encoded metallo-β-lactamase.

## Supplementary Materials

The following supporting information can be downloaded at https://www.mdpi.com/article/10.3390/pathogens14080777/s1: Supplementary Table S1. Detailed search strategies used in each resource (as of 23 June 2025). Supplementary Table S2. PRISMA 2020 for the abstract reporting checklist. Supplementary Table S3. PRISMA 2020 for the main article text reporting checklist.

## Abbreviations

ALT	alanine aminotransferase
AmpC	AmpC β-lactamase enzyme
AST	aspartate aminotransferase
CFU	colony-forming units
cIAI	complicated intra-abdominal infection
CLSI	Clinical and Laboratory Standards Institute
cUTIs	complicated urinary tract infections
DOI	digital object identifier
ELF	epithelial lining fluid
EMA	European Medicines Agency
ESBL	extended-spectrum β-lactamase
EUCAST	European Committee on Antimicrobial Susceptibility Testing
FDA	Food and Drug Administration
HAP	hospital-acquired pneumonia
KPC	*Klebsiella pneumoniae* carbapenemase
MBL	metallo-β-lactamase
MIC	minimum inhibitory concentration
NDM	New Delhi metallo-β-lactamase
PD	pharmacodynamic
PK/PD	pharmacokinetic/pharmacodynamic
PK	pharmacokinetic
PRISMA	Preferred Reporting Items for Systematic Reviews and Meta-Analyses
VAP	including ventilator-associated pneumonia
VIM	Verona integron-encoded metallo-β-lactamase
XDR	extensively drug-resistant
β-lactams	Beta-lactams

## References

[1] Papp-Wallace K.M. (2019). The latest advances in β-lactam/β-lactamase inhibitor combinations for the treatment of Gram-negative bacterial infections. Expert Opin. Pharmacother..

[2] Bergan T. (1984). Pharmacokinetics of beta-lactam antibiotics. Scand. J. Infect. Dis. Suppl..

[3] Sargianou M., Stathopoulos P., Vrysis C., Tzvetanova I.D., Falagas M.E. (2025). New β-Lactam/β-Lactamase Inhibitor Combination Antibiotics. Pathogens.

[4] Bhowmick T., Canton R., Pea F., Quevedo J., Santerre Henriksen A., Timsit J.-F., Kaye K.S. (2025). Cefepime-enmetazobactam: First approved cefepime-β- lactamase inhibitor combination for multi-drug resistant Enterobacterales. Future Microbiol..

[5] U.S. Food and Drug Administration Exblifep® (2024). (Cefepime and Enmetazobactam) for Injection, for Intravenous Use: Highlights of Prescribing Information. https://www.accessdata.fda.gov/drugsatfda_docs/label/2024/216165s000lbl.pdf.

[6] (2024). Exblifep European Medicines Agency (EMA). https://www.ema.europa.eu/en/medicines/human/EPAR/exblifep.

[7] Johnson A., McEntee L., Farrington N., Kolamunnage-Dona R., Franzoni S., Vezzelli A., Massimiliano M., Knechtle P., Belley A., Dane A. (2020). Pharmacodynamics of Cefepime Combined with the Novel Extended-Spectrum-β-Lactamase (ESBL) Inhibitor Enmetazobactam for Murine Pneumonia Caused by ESBL-Producing Klebsiella pneumoniae. Antimicrob. Agents Chemother..

[8] Das S., Fitzgerald R., Ullah A., Bula M., Collins A.M., Mitsi E., Reine J., Hill H., Rylance J., Ferreira D.M. (2020). Intrapulmonary Pharmacokinetics of Cefepime and Enmetazobactam in Healthy Volunteers: Towards New Treatments for Nosocomial Pneumonia. Antimicrob. Agents Chemother..

[9] Darlow C.A., Hope W., Dubey V. (2025). Cefepime/Enmetazobactam: A microbiological, pharmacokinetic, pharmacodynamic, and clinical evaluation. Expert Opin. Drug Metab. Toxicol..

[10] Clinical and Laboratory Standards Institute (CLSI) (2024). Performance Standards for Antimicrobial Susceptibility Testing.

[11] Page M.J., McKenzie J.E., Bossuyt P.M., Boutron I., Hoffmann T.C., Mulrow C.D., Shamseer L., Tetzlaff J.M., Akl E.A., Brennan S.E. (2021). The PRISMA 2020 statement: An updated guideline for reporting systematic reviews. BMJ.

[12] Belley A., Morrissey I., Hawser S., Kothari N., Knechtle P. (2021). Third-generation cephalosporin resistance in clinical isolates of Enterobacterales collected between 2016–2018 from USA and Europe: Genotypic analysis of β-lactamases and comparative in vitro activity of cefepime/enmetazobactam. J. Glob. Antimicrob. Resist..

[13] Bonnin R.A., Jeannot K., Santerre Henriksen A., Quevedo J., Dortet L. (2025). In vitro activity of cefepime-enmetazobactam on carbapenem-resistant Gram negatives. Clin. Microbiol. Infect..

[14] Carlos Vázquez-Ucha J., Lasarte-Monterrubio C., Guijarro-Sánchez P., Oviaño M., Álvarez-Fraga L., Alonso-García I., Arca-Suárez J., Bou G., Beceiro A., Instituto de Investigación Biomédica de A Coruña La Corogne (2022). Assessment of Activity and Resistance Mechanisms to Cefepime in Combination with the Novel b-Lactamase Inhibitors Zidebactam, Taniborbactam, and Enmetazobactam Against a Multicenter Collection of Carbapenemase-Producing Enterobacterales. https://agris.fao.org/search/en/providers/122439/records/67dad517677d8be0233bfede.

[15] Emeraud C., De Swardt H., Bernabeu S., Latour L., Pages A., Ronsin S., Bonnin R.A., Dortet L. (2025). Comparative evaluation of disc diffusion and Liofilchem^TM^ MTS strip methods with broth microdilution for cefepime/enmetazobactam susceptibility testing. J. Antimicrob. Chemother..

[16] Kadry A.A., El-Antrawy M.A., El-Ganiny A.M. (2022). Management of clinical infections of Escherichia coli by new β-lactam/β-lactamase inhibitor combinations. Iran. J. Microbiol..

[17] Lee Y.-L., Ko W.-C., Lee W.-S., Lu P.-L., Chen Y.-H., Cheng S.-H., Lu M.-C., Lin C.-Y., Wu T.-S., Yen M.-Y. (2021). In-vitro activity of cefiderocol, cefepime/zidebactam, cefepime/enmetazobactam, omadacycline, eravacycline and other comparative agents against carbapenem-nonsusceptible Enterobacterales: Results from the Surveillance of Multicenter Antimicrobial Resistance in Taiwan (SMART) in 2017–2020. Int. J. Antimicrob. Agents.

[18] Liu P.-Y., Ko W.-C., Lee W.-S., Lu P.-L., Chen Y.-H., Cheng S.-H., Lu M.-C., Lin C.-Y., Wu T.-S., Yen M.-Y. (2022). In vitro activity of cefiderocol, cefepime/enmetazobactam, cefepime/zidebactam, eravacycline, omadacycline, and other comparative agents against carbapenem-non-susceptible Pseudomonas aeruginosa and Acinetobacter baumannii isolates associated from bloodstream infection in Taiwan between 2018–2020. J. Microbiol. Immunol. Infect..

[19] Morrissey I., Hawser S., Kothari N., Dunkel N., Quevedo J., Belley A., Henriksen A.S., Attwood M. (2024). Evaluation of the activity of cefepime/enmetazobactam against Enterobacterales bacteria collected in Europe from 2019 to 2021, including third-generation cephalosporin-resistant isolates. J. Glob. Antimicrob. Resist..

[20] Morrissey I., Magnet S., Hawser S., Shapiro S., Knechtle P. (2019). In vitro Activity of Cefepime-Enmetazobactam against Gram-Negative Isolates Collected from U.S. and European Hospitals during 2014–2015. Antimicrob. Agents Chemother..

[21] Tselepis L., Langley G.W., Aboklaish A.F., Widlake E., Jackson D.E., Walsh T.R., Schofield C.J., Brem J., Tyrrell J.M. (2020). In vitro efficacy of imipenem-relebactam and cefepime-AAI101 against a global collection of ESBL-positive and carbapenemase-producing Enterobacteriaceae. Int. J. Antimicrob. Agents.

[22] Vasquez-Ponce F., Dantas K., Becerra J., Melocco G., Esposito F., Cardoso B., Rodrigues L., Lima K., de Lima A.V., Sellera F.P. (2022). Detecting KPC-2 and NDM-1 Coexpression in Klebsiella pneumoniae Complex from Human and Animal Hosts in South America. Microbiol. Spectr..

[23] Falagas M.E., Kontogiannis D.S., Zidrou M., Filippou C., Tansarli G.S. (2025). Global Epidemiology and Antimicrobial Resistance of Metallo-β-Lactamase (MBL)-Producing Acinetobacter Clinical Isolates: A Systematic Review. Pathogens.

[24] Lang P.A., Raj R., Tumber A., Lohans C.T., Rabe P., Robinson C.V., Brem J., Schofield C.J. (2022). Studies on enmetazobactam clarify mechanisms of widely used β-lactamase inhibitors. Proc. Natl. Acad. Sci. USA.

[25] Bernhard F., Odedra R., Sordello S., Cardin R., Franzoni S., Charrier C., Belley A., Warn P., Machacek M., Knechtle P. (2020). Pharmacokinetics-pharmacodynamics of enmetazobactam combined with cefepime in a neutropenic murine thigh infection model. Antimicrob. Agents Chemother..

[26] Karaiskos I., Giamarellou H. (2020). Carbapenem-Sparing Strategies for ESBL Producers: When and How. Antibiotics.

[27] Falagas M.E., Kastoris A.C., Kapaskelis A.M., Karageorgopoulos D.E. (2010). Fosfomycin for the treatment of multidrug-resistant, including extended-spectrum beta-lactamase producing, Enterobacteriaceae infections: A systematic review. Lancet Infect. Dis..

[28] Falagas M.E., Vouloumanou E.K., Samonis G., Vardakas K.Z. (2016). Fosfomycin. Clin. Microbiol. Rev..

[29] Carmeli Y., Cisneros J.M., Paul M., Daikos G.L., Wang M., Torre-Cisneros J., Singer G., Titov I., Gumenchuk I., Zhao Y. (2025). Aztreonam–avibactam versus meropenem for the treatment of serious infections caused by Gram-negative bacteria (REVISIT): A descriptive, multinational, open-label, phase 3, randomised trial. Lancet Infect. Dis..

[30] Torres A., Zhong N., Pachl J., Timsit J.-F., Kollef M., Chen Z., Song J., Taylor D., Laud P.J., Stone G.G. (2018). Ceftazidime-avibactam versus meropenem in nosocomial pneumonia, including ventilator-associated pneumonia (REPROVE): A randomised, double-blind, phase 3 non-inferiority trial. Lancet Infect. Dis..

[31] Solomkin J., Hershberger E., Miller B., Popejoy M., Friedland I., Steenbergen J., Yoon M., Collins S., Yuan G., Barie P.S. (2015). Ceftolozane/Tazobactam Plus Metronidazole for Complicated Intra-abdominal Infections in an Era of Multidrug Resistance: Results from a Randomized, Double-Blind, Phase 3 Trial (ASPECT-cIAI). Clin. Infect. Dis..

[32] Kollef M.H., Nováček M., Kivistik Ü., Réa-Neto Á., Shime N., Martin-Loeches I., Timsit J.-F., Wunderink R.G., Bruno C.J., Huntington J.A. (2019). Ceftolozane–tazobactam versus meropenem for treatment of nosocomial pneumonia (ASPECT-NP): A randomised, controlled, double-blind, phase 3, non-inferiority trial. Lancet Infect. Dis..

[33] Sahni R.D., Balaji V., Varghese R., John J., Tansarli G.S., Falagas M.E. (2013). Evaluation of fosfomycin activity against uropathogens in a fosfomycin-naive population in South India: A prospective study. Future Microbiol..

[34] Falagas M.E., Maraki S., Karageorgopoulos D.E., Kastoris A.C., Mavromanolakis E., Samonis G. (2010). Antimicrobial susceptibility of multidrug-resistant (MDR) and extensively drug-resistant (XDR) Enterobacteriaceae isolates to fosfomycin. Int. J. Antimicrob. Agents.

[35] Katsarou A., Stathopoulos P., Tzvetanova I.D., Asimotou C.-M., Falagas M.E. (2025). β-Lactam/β-Lactamase Inhibitor Combination Antibiotics Under Development. Pathogens.

[36] Bassetti M., Giacobbe D.R. (2024). Cefepime-taniborbactam and CERTAIN-1: Can we treat carbapenem-resistant infections?. Med.

[37] Roach E.J., Uehara T., Daigle D.M., Six D.A., Khursigara C.M. (2021). The Next-Generation β-Lactamase Inhibitor Taniborbactam Restores the Morphological Effects of Cefepime in KPC-Producing *Escherichia coli*. Microbiol. Spectr..

[38] Moya B., Barcelo I.M., Bhagwat S., Patel M., Bou G., Papp-Wallace K.M., Bonomo R.A., Oliver A. (2017). WCK 5107 (Zidebactam) and WCK 5153 Are Novel Inhibitors of PBP2 Showing Potent “β-Lactam Enhancer” Activity against Pseudomonas aeruginosa, Including Multidrug-Resistant Metallo-β-Lactamase-Producing High-Risk Clones. Antimicrob. Agents Chemother..

[39] Wockhardt (2025). A Phase 3, Randomized, Double-Blind, Multicenter, Comparative Study to Determine the Efficacy and Safety of Cefepime-Zidebactam vs. Meropenem in the Treatment of Complicated Urinary Tract Infection or Acute Pyelonephritis in Adults.

[40] Romanos L.T., Kontogiannis D.S., Tsiampali C., Tzvetanova I.D., Falagas M.E. (2025). Antibiotics and non-traditional antimicrobial agents for Pseudomonas aeruginosa in clinical phases 1, 2, and 3 trials. Expert Opin. Investig. Drugs.

[41] Kaye K.S., Belley A., Barth P., Lahlou O., Knechtle P., Motta P., Velicitat P. (2022). Effect of Cefepime/Enmetazobactam vs Piperacillin/Tazobactam on Clinical Cure and Microbiological Eradication in Patients With Complicated Urinary Tract Infection or Acute Pyelonephritis: A Randomized Clinical Trial. JAMA.

[42] Allecra (2019). A Phase 1, Open-Label, Single-Dose Study to Assess the Mass Balance, Pharmacokinetics and Metabolism of Intravenously Administered 14C-AAI101 in Healthy Male Subjects.

[43] Allecra (2022). Phase 1, Open-Label, Parallel Group, Single-Dose Study to Evaluate the Pharmacokinetics, Safety and Tolerability of AAI101 With Cefepime in Subjects With Varying Degrees of Renal Function.

[44] Allecra (2020). A Phase I Open-Label, Single-Centre Study to Assess the Concentration of AAI101 and Cefepime in Epithelial Lining Fluid and Plasma in Healthy Volunteers.

[45] Allecra (2018). Phase I, Single and Multiple Ascending Dose Study to Investigate the Safety, Tolerability and Pharmacokinetics of AAI101 Administered Intravenously Alone or in Combination With Piperacillin or Cefepime to Healthy Adult Subjects.

[46] Allecra (2018). Randomized, Double-Blind, Multi-Center Study of Cefepime/AAI101 in Hospitalized Adults with Complicated Urinary Tract Infections, Including Acute Pyelonephritis.

[47] Allecra (2024). Single Group Phase 2 Study to Investigate Pharmacokinetics, Safety and Tolerability of Cefepime-Enmetazobactam Administered by IV over 2 Hr in Male or Female Participants from Birth to Less Than 18 Years of Age Hospitalised with cUTI.

[48] Albac S., Anzala N., Chavanet P., Dunkel N., Quevedo J., Santerre Henriksen A., Croisier D. (2024). In vivo efficacy of enmetazobactam combined with cefepime in a murine pneumonia model induced by OXA-48-producing Klebsiella pneumoniae. Microbiol. Spectr..

