# Supplementary material for: Resistance of Gram-Negative Bacteria to Cefepime-Enmetazobactam: A Systematic Review
Source: Pathogens. 2025 Aug 6;14(8):777. doi: 10.3390/pathogens14080777 (PMC12389057; doi:10.3390/pathogens14080777)
Supplement: Supplementary file 1 [file pathogens-14-00777-s001.zip › 1. Supplemetnary Table 1-3796417.docx]

**Supplementary Table 1. Detailed search strategies used in each resource as of 23 June 2025.**

| Resource | Search string | Coverage (years) | Results |
| --- | --- | --- | --- |
| Embase | ("cefepime-enmetazobactam" OR (cefepime AND enmetazobactam)) AND ("antibiotic resistance" OR resistance OR "non-susceptibility" OR "non susceptibility" OR nonsusceptibility OR "reduced susceptibility") AND (MIC OR "minimum inhibitory concentration" OR "disc diffusion") | 1947 –present | 25 |
| PubMed | ("cefepime-enmetazobactam" OR (cefepime AND enmetazobactam)) AND ("antibiotic resistance" OR resistance OR "non-susceptibility" OR "non susceptibility" OR nonsusceptibility OR "reduced susceptibility") AND (MIC OR "minimum inhibitory concentration" OR "disc diffusion") | 1946 –present | 18 |
| Scopus | ("cefepime-enmetazobactam" OR (cefepime AND enmetazobactam)) AND ("antibiotic resistance" OR resistance OR "non-susceptibility" OR "non susceptibility" OR nonsusceptibility OR "reduced susceptibility") AND (MIC OR "minimum inhibitory concentration" OR "disc diffusion") | 1966 –present | 19 |
| Web of Science | ("cefepime-enmetazobactam" OR (cefepime AND enmetazobactam)) AND ("antibiotic resistance" OR resistance OR "non-susceptibility" OR "non susceptibility" OR nonsusceptibility OR "reduced susceptibility") AND (MIC OR "minimum inhibitory concentration" OR "disc diffusion") | 1900 – present | 9 |
